# Hummers’ method-assisted liquid-phase exfoliation enables the fabrication of few-layer borophene from bulk boron

**DOI:** 10.55730/1300-0527.3659

**Published:** 2024-02-22

**Authors:** Mehmet Semih BİNGÖL, Mehmet YILMAZ, Ahmet Emre KASAPOĞLU

**Affiliations:** 1 East Anatolia High Technology Application and Research Center (DAYTAM), Atatürk University, Erzurum, Turkiye; 2 Department of Chemical Engineering, Faculty of Engineering, Atatürk University, Erzurum, Turkiye

**Keywords:** Two-dimensional materials, few-layer borophene, sonication, liquid-phase exfoliation, elemental boron, Hummers’ method

## Abstract

The fabrication of few-layer borophene (BP) from bulk boron (b-B) is of great importance and still a scientific challenge due to the complex structure and crystallinity of b-B. Herein, we propose a novel technique to prepare a few-layer BP on a large scale with a large lateral size in a well-controlled manner. For this, we employed the Hummers’ method-assisted liquid-phase exfoliation. In the first step, the chemical exfoliation of the b-B as a precursor was performed by the modified Hummers’ method. After chemical exfoliation, mechanical delamination was employed by using an immersion sonicator. Finally, BP sheets were collected with dimensions ranging from several hundred nanometers to a few micrometers and an average thickness of 4.2 nm. We envision that the proposed low-cost, flexible, and large-scale production method will provide unique advantages for the application of few-layer BP in the realization of high-performance electronics, optoelectronics, flexible devices, sensing systems, energy conversion, and storage devices.

## 1. Introduction

In the last two decades, two-dimensional (2D) materials including graphene, black phosphorus, MXene, hexagonal boron nitride, and transition metal dichalcogenides have gained remarkable attention [1]. In comparison to their bulk counterparts, these materials represent extraordinary optical, electronic, and phonon properties due to their novel structures [2–7]. These characteristics of 2D materials enabled their potential application in various fields such as electronics, optoelectronics, flexible devices, sensing, energy conversion, and storage [8–16]. Despite the recent progress in the fabrication and application of these structures, 2D materials with novel structures and characteristics are still highly demanded in applied research.

Recently, atomically thin boron (B) (named borophene, BP) was discovered and proposed as an ideal alternative to other traditional 2D materials [17–19]. The BP has been employed in many applications due to its tunable band gap, high chemical stability, high carrier mobility, superconductivity, and super hardness [14,20–25]. Although BP has attracted remarkable attention, there were limited experimental studies on the fabrication of these 2D materials. In recent years, vapor-based fabrication methods have been utilized with different approaches. For instance, B sheets and BP have been deposited onto the silver surfaces under ultra-high-vacuum conditions [17,18]. Similarly, Tai et al. have shown the preparation of atomically thin B films onto the Cu foils through the chemical vapor deposition technique [19]. Also, B sheets have been prepared by the decomposition of diborane under reduced pressure in the report by Xu et al. [23]. However, the high cost of ultra-high-vacuum fabrication devices, insufficiencies in large-scale fabrication, deteriorations in the quality of sheets, and the high number of process steps have arisen as limitations of these techniques and restrict their potential applications [26]. Therefore, the fabrication of a few-layer BP on a large scale with high quality is of great importance and highly requested. So far, sonication-assisted liquid-phase exfoliation and, chemical exfoliation as alternative methods have been employed for the fabrication of 2D materials [27–31]. In these approaches, the complexity and diversity of structures and crystallinity of b-B endanger the formation of the few-layer BP in a well-controlled manner. Thus, a limited number of works have been performed to fabricate BP through liquid-phase exfoliation. Xie et al. could synthesize B nanosheets with an average thickness of 1.8 nm and lateral size of a few hundred nanometers by using liquid-phase exfoliation combined with sonication [1]. Recently, similar results were obtained by the reports of the Teo group [4] and the Vinyl group [32]. It is obvious that developing novel and efficient protocols to fabricate few-layer BP with large lateral size in a well-controlled manner is a scientific challenge and will provide unique opportunities to this research field.

Herein, we propose, for the first time, the Hummers’ method-assisted liquid-phase exfoliation for the fabrication of few-layer BP from the b-B. In the first step, the chemical exfoliation of the b-B as a precursor was performed by the modified Hummers’ method which was similar to the graphite exfoliation. After chemical exfoliation, we employed mechanical delamination by using an immersion sonicator in DMF for 12 h. Finally, we collected BP sheets with dimensions ranging from several hundred nanometers to a few micrometers and an average thickness of 4.2 nm. We envision that the proposed low-cost, flexible, and large-scale production method will pave the way for the application of few-layer BP in the realization of high-performance electronics, optoelectronics, flexible devices, sensing systems, energy conversion, and storage devices.

## 2. Materials and methods

### 2.1 Materials

b-B (99% pure and < 1 micron) was obtained from J/K. Potassium permanganate, H2SO4, dimethylformamide (DMF), and hydrogen peroxide were purchased from Merck.

### 2.2 Preparation of 2D BP material

In this study, 2D BP material was prepared through two sequential procedures: Chemical exfoliation and mechanical delamination. In the first step, the chemical exfoliation of the b-B as a precursor was performed by the modified Hummers’ method which was similar to the graphite exfoliation. For this, 750 mg of b-B was mixed with 3000 mg of potassium permanganate in a three-neck flask and then 60 mL of sulfuric acid (96%) and 7 mL of H3PO4 were added slowly. The resultant mixture was stirred at 0 °C for 2 h. Afterward, the mixture was heated to 40 °C and kept in this condition for an additional 2 h. The heater was turned off and the mixture was cooled to ambient conditions and transferred into an ice bath. At this stage, 200 mL of hydrogen peroxide was added to the mixture and the resultant product (c-BP) was purified by washing many times with distilled water through centrifugation.

After chemical exfoliation, we employed mechanical delamination by using an immersion sonicator (75W). For this, c-BP was sonicated in DMF for 12 h. Afterward, the resultant product (m-BP) was separated from the solution using centrifugation.

### 2.3 Characterization of materials

The morphology of materials was determined by utilizing transmission electron microscopy (TEM, Hitachi HighTech HT7700, Japan) at 120 kV and scanning electron microscopy (SEM, Zeiss Gemini sigma 300). XRD patterns were collected by using PANalytical Empyrean diffractometer with the following operating settings: scan rate- 4°/min, operating voltage −45 kV, operating current −40 mA, scan range 10–90°, and, scan step 0.013. X-ray photoelectron spectroscopy (XPS, Specs Flexmod) was employed to obtain the atomic and molecular structure of the products. In a similar vein, Raman spectra of samples were obtained through a WITec alpha 300 R Micro-Raman spectrometer with a 532 nm laser source and 0.3 mW laser power. Also, the zeta potential and particle size distribution of the samples were recorded by employing a Malvern Zetasizer Nanozs model at the neutral pH of the dispersions at 25 °C. Before each measurement, the samples were well-dispersed in distilled water through a sonicator for 5 min. The AFM surface topography image of m-BP was performed through a Hitachi 5100N model. The scanning area was 10 μm × 10 μm.

## 3. Results and discussion

**Figure 1:**
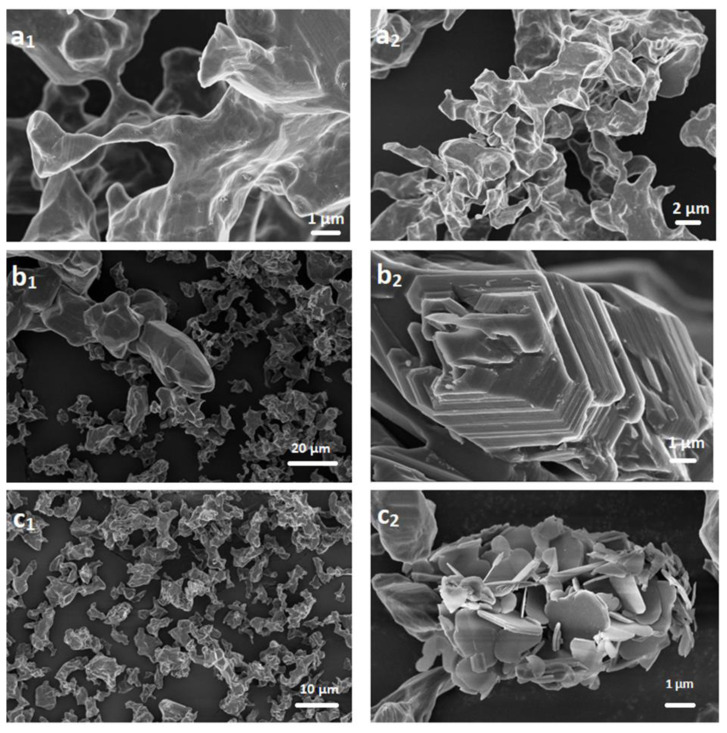
SEM images of b-B (a), c-BP (b), and, m-BP (c) at different magnifications.

**Figure 2:**
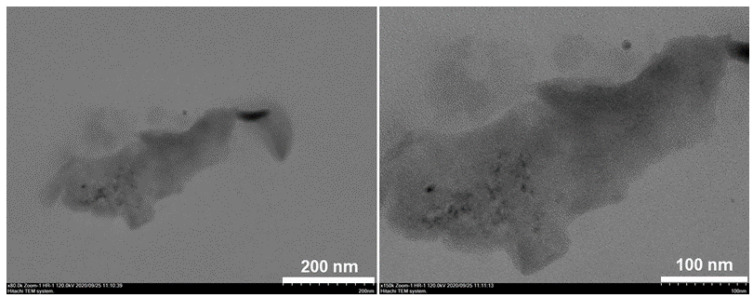
TEM images of m-BP at different magnifications.

**Figure 3:**
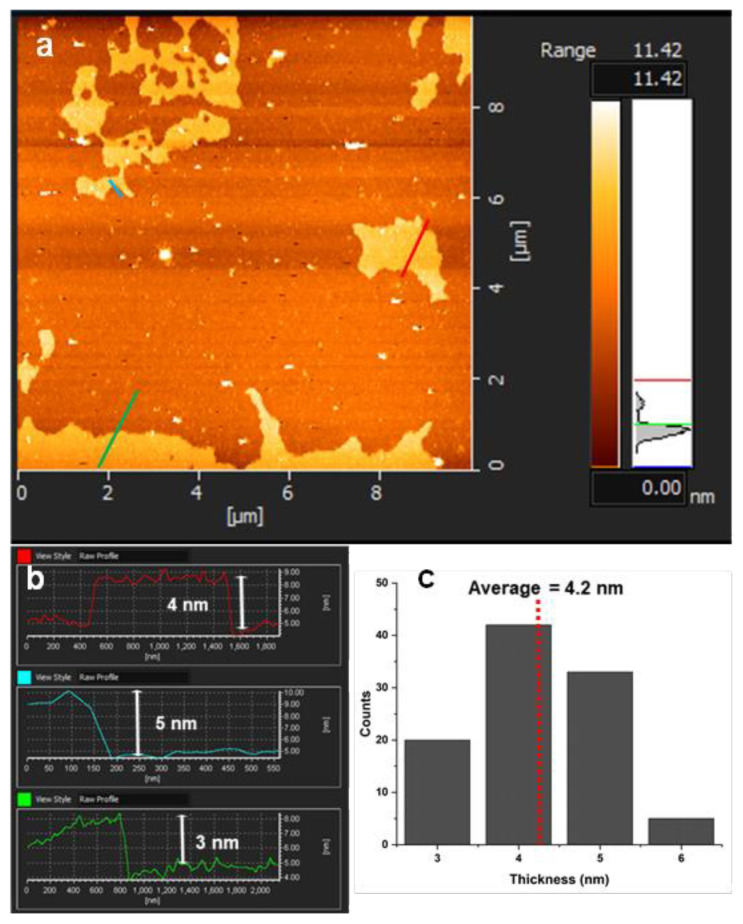
2D AFM images (a), height change of the red, turquoise, and green line (b), and thickness distribution (c) of few-layer m-BP.

Firstly, we evaluated the final morphology of b-B, c-BP, and, m-BP through a detailed SEM analysis. For each material, representative SEM images at different magnifications are summarized in Figure 1. For the case of pristine b-B (Figure 1a), we detected the powders of b-B with a size distribution ranging from a few hundred nanometers to a few micrometers. After the chemical exfoliation of the b-B through the modified Hummers’ method (Figure 1b), the stacked layers of B were noticed. It seems that the employment of the Hummers method resulted in the emergence of the layers of B with high integration. However, after mechanical delamination by using an immersion sonicator (Figure 1c), a dramatic change was detected in the final morphology of the resultant product. The mechanical delamination procedure led to the disintegration and exfoliation of stacked layers and the formation of B sheets with dimensions ranging from several hundred nanometers to a few micrometers. To furtherly confirm the few-layer thin m-BP sheets, we collected some representative TEM images (Figure 2). We detected that various small numbers of atomically thin m-BP sheets with a lateral size ranging from lower than 100 nm to a few micrometers were detected in the sample. 2D AFM images of m-BP (Figure 3) strongly support the TEM observations. The existence of a few-layer m-BP was clearly detected from representative AFM topographic images (Figure 3a). Also, the height distribution analysis of the sheets indicated that their thickness was in the range of 3–6 nm with an average of 4.2 nm (Figures 3b–3c). When the literature was examined, it was seen that the distance between the layers of the obtained borophenes was 0.5 nm. Based on this, it has been understood that the borophenes we prepared are seperated into 8 or 9 layers [4,33].

**Figure 4:**
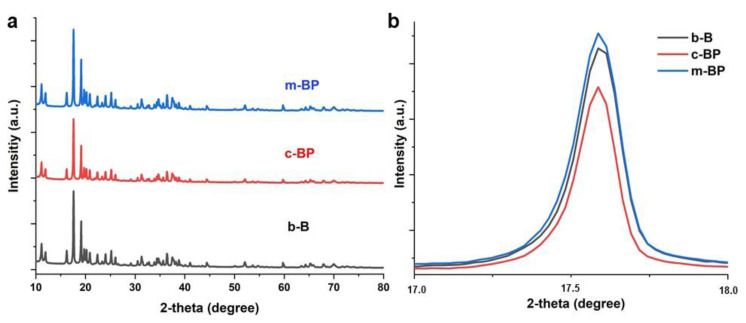
XRD patterns b-B, c-BP, and, m-BP samples (a) and XRD specific peak broadening (b) after B delamination.

After morphological characterization, we utilized some spectroscopic analyses to investigate the characteristics of each product. For this, firstly, we collected the XRD patterns of the samples (Figure 4a). For the case of b-B, we detected that the XRD pattern was highly compatible with pure B and all the diffraction peaks were assigned to the β-rhombohedral B (JCPDF 00-031-0207) [4,16]. No significant change was observed in the XRD patterns of c-BP (b) and, m-BP indicating that the crystalline structure of B was mainly conserved after chemical exfoliation and, mechanical delamination. However, the detailed analysis of diffraction peaks may provide valuable information to evaluate the minor changes in crystal structures after delamination. For instance, the major peak at 17.6o led to a remarkable peak broadening (Figure 4b) after delamination suggesting the emergence of BP structure [16].

**Figure 5:**
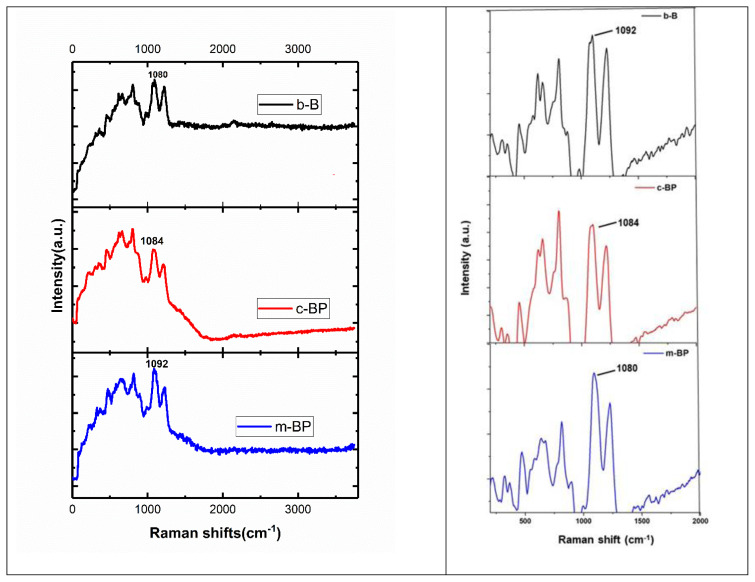
Raman spectra of b-B, c-BP, and m-BP.

An efficient way to describe the atomic bonding in materials is with Raman spectroscopy. More typical Raman peaks for borophene should be noticed due to anisotropy, no equal symmetry axes, and atomic displacements toward the atomic ridges of borophene. We also collected the Raman spectra of each sample to determine the molecular structure changes (Figure 5). Similar peaks were seen in all three cases. For the case of b-B, we detected Raman vibration peaks at 454, 651, 806 1092, and 1225 cm-1 indicating the nature of the β-rhombohedral B with different lattice structures [16,34,35]. Compared to Raman bands of b-B, the remarkable changes especially peak maxima shifts were observed after chemical exfoliation and, mechanical delamination. For instance, A1g Raman mode at 1092 cm-1 was shifted to 1084 and 1081 cm-1 for the case of c-BP and m-BP, respectively. A similar trend was noticed for the other Raman bands. The differences in Raman bands may be attributed to highly-expected molecular changes in b-B and prepared BP products.

**Figure 6:**
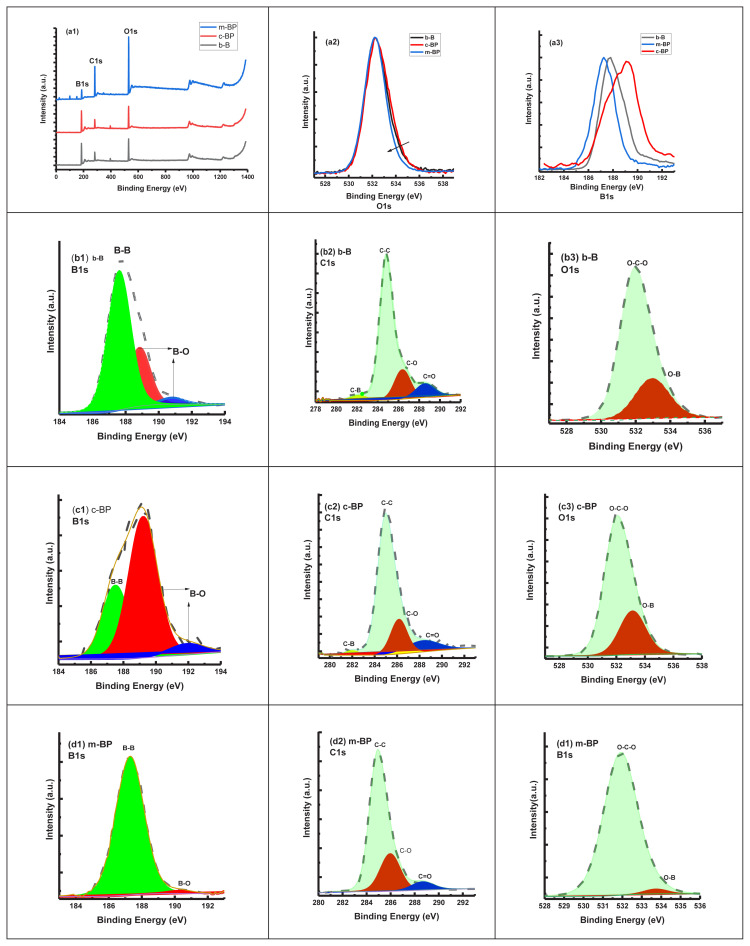
XPS spectra (survey) (a1) and short scan XPS B1s (b1, c1, and d1), C1s (b2, c2, and d2), and O1s (b3, c3, and d3) for b-B, c-BP, and m-BP and comparison of O1s (a2) and B1s (a3).

We also utilized the XPS analysis to furtherly investigate the surface composition and chemical state of the B-based materials before and after the sonication-assisted Hummers method as liquid-phase exfoliation. Figure 6 and Table summarize the XPS analysis of b-B, c-BP, and m-BP. The XPS survey scan spectra of each material (Figure 6a1) ranging from 0 to 800 eV indicated that the main peaks were assigned to B, C, and O. In general, there was no observed significant change in the B content indicating almost no change in the surface compositions after chemical exfoliation and, mechanical delamination [16]. After the XPS survey scan, we performed a detailed short scan of B1s, C1s, and O1s of the relevant materials. The high-resolution B1s spectra consist of three peaks centered at 190.81, 188.87, and 187.61 eV (Figures 6b1, 6c1, and 6d1). The prominent peak at 187.61 was assigned to a B − B bond, which is highly consistent with the b-B. However, the higher binding energy peaks at 188.87 and 190.81 eV indicate the oxidation of b-B [36–38]. For the case of m-BP, we detected the highest B−B bond with an 86.68% atomic ratio (see Table). It is noted that the main binding energy peaks for the B component are slightly shifted (187.3 eV) in comparison to b-B. The B-B component ratio of b-B was decreased after the chemical process. Therefore, the ratio of B-O was increased. Here, this result may be attributed to the increase of oxidation after the chemical process. However, after the mechanical process (i.e. sonication), it was found that the B-B component ratio increased dramatically relative to b-B.

The high-resolution C1s spectra of the b-B, c-BP, and m-BP (Figures 6b2, 6c2, and 6d2) can be deconvoluted into four components at around 281.98, 284.87, 286.47, and 288.67 eV, which were assigned to the C−B, C−C, C-C−O, and C=O bonds, respectively. The further analysis of the C1s spectrum of the b-B and c-BP showed that the B-C bond with an energy value of 281.98 eV was not detected after the mechanical process. This observation indicates that most of the B atoms remain intact after the mechanical process.

From detailed XPS spectra analysis, it was concluded that after the mechanical process, almost no C-B bond remained (see Table). Finally, we compared the O1s (a2) and B1s spectra of each sample in Figures 6a2 and 6a3 in-depth. We observed that there was a narrowing in the direction of the arrow in the O1s spectrum (Figure 6a2). Therefore, the intensity of B-O bonds decreased remarkably. Besides, the B-B bond in the B1s spectra was more apparent after the mechanical process.

**Figure 7:**
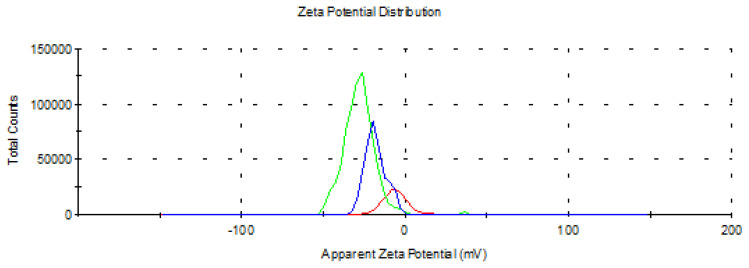
Zeta potential distribution of b-B, c-BP, and m-BP.

**Figure 8:**
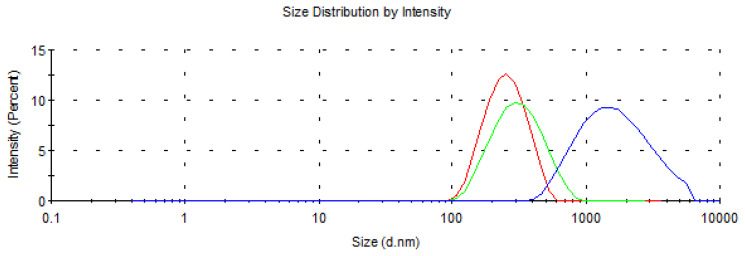
Zeta sizer distribution of b-B, c-BP, and m-BP.

Finally, we performed a zeta potential/size analysis of b-B, c-BP, and m-BP (Figure 7–8). The mean scores of zeta potential were found to be −6.5, −24.0, and −29.8 mV for b-B, c-BP, and m-BP, respectively (Figure 7). Since zeta potential is an indicator of the stability of a suspension, we can obviously state that the stability of b-B was remarkably increased after chemical exfoliation and, mechanical delamination. When the b-B was exfoliated at each stage, the relevant zeta potential was increased gradually. As the particles’ size decreased, particle mobility increased, leading to an increase in the zeta potential.

The mean scores of particle size distribution are 1335, 622 and, 540 nm for b-B, c-BP, and m-BP, respectively (Figure 8). The employment of the chemical and mechanical processes led to a decrease in particle size at each stage. The size distribution of b-B created a broad peak indicating particles of several different sizes. However, after the chemical and mechanical process, both the average size and the width of the peaks decreased significantly. This result was highly correlated with SEM, AFM, and TEM data.

## 4. Conclusion

In short, we developed an easy, low-cost, and flexible approach for the liquid-phase exfoliation of b-B by employing the modified Hummers’ method and sonication as the mechanical method. After the chemical and mechanical process, we detected the disintegration and exfoliation of stacked layers and the formation of B sheets with dimensions ranging from several hundred nanometers to a few micrometers and an average thickness of 4.2 nm. XRD patterns of c-BP and m-BP showed that the crystalline structure of B was mainly conserved after chemical exfoliation and, mechanical delamination. The detailed XPS analysis indicated minor changes in the surface composition and chemical state of the B-based materials. Also, zeta potential/sizer distributions detected the increase in potential and decrease in the size suggesting the high stability and the disintegration and exfoliation of stacked layers and the formation of B sheets after the proposed approach. We strongly believe that the production of few-layer B on a large scale will pave the way for potential applications including flexible electronics, energy storage devices, and, optoelectronics.

## Figures and Tables

**Table:** The quantitative XPS atomic ratio analysis of b-B, c-BP, and m-BP.

Sample	B1s atomic ratio (%)			C1s atomic ratio (%)			
	B-B	BO	BO (B_2_O3)	C-B	C-C	C-O	C=O
b-B	70.28	20.26	3.47	2.89	70.41	16.54	10.16
c-BP	30.06	64.10	5.84	2.12	67.21	18.71	11.96
m-BP	86.68	12.98	-----	-----	68.00	25.65	6.35
